# Supplementary material for: Pore Structure and Properties of PEEK Hollow Fiber Membranes: Influence of the Phase Structure Evolution of PEEK/PEI Composite
Source: Polymers (Basel). 2019 Aug 26;11(9):1398. doi: 10.3390/polym11091398 (PMC6780917; doi:10.3390/polym11091398)
Supplement: Supplementary file 1 [file polymers-11-01398-s001.pdf]

Table S1. Mass loss rate of the fibers with different extraction times.

extraction time（h）	Pre-extraction mass (g)	After extraction mass (g)	Mass loss rate (%)
3		0.172	52.5
6		0.165	54.4
9	0.362	0.160	55.8
12		0.162	55.3
24		0.159	56.0

[1]When PEI is completely extracted, the theoretical mass loss rate is 60%.

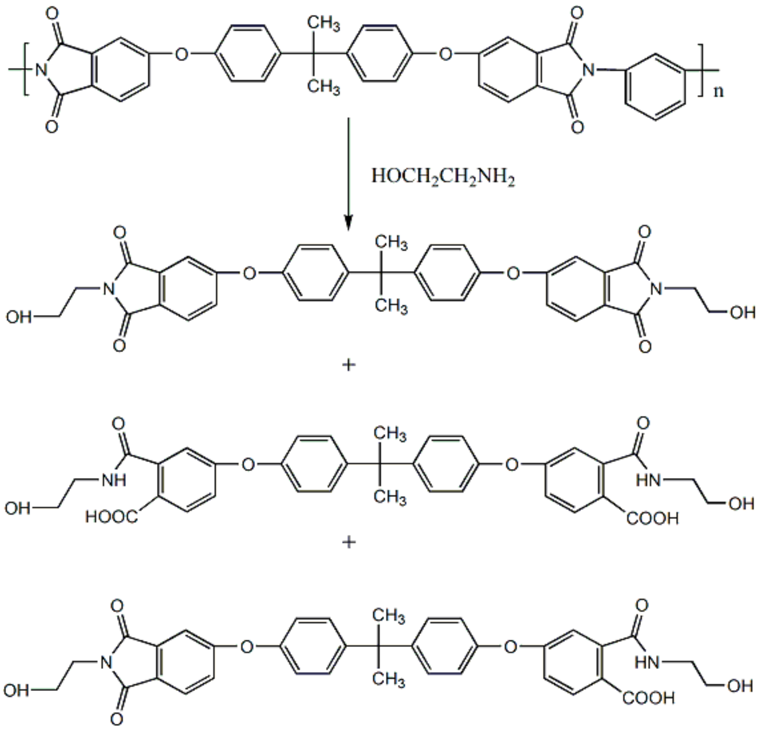

Figure S1. Decomposition reaction mechanism of PEI in the extraction solution.

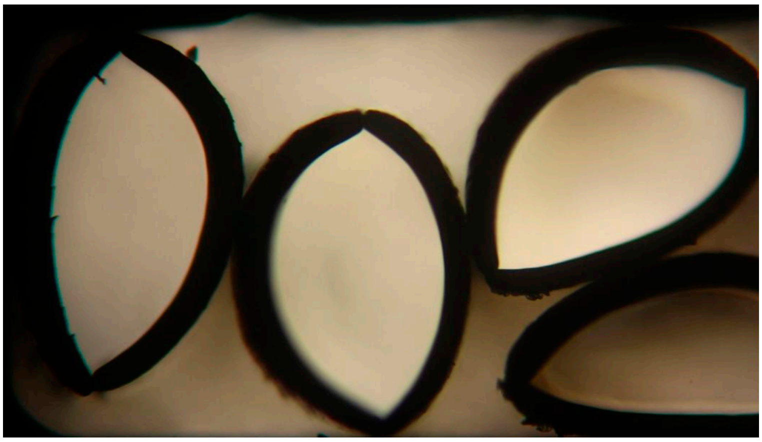

Figure S2. The cross-section of PEEK/PEI hollow fiber by optical microscope (100×).
